# Refractive Lens Exchange Surgery in Early-Onset High Myopia Patients With Partial Cataract

**DOI:** 10.3389/fmed.2022.739197

**Published:** 2022-04-15

**Authors:** Xi-Fang Zhang, Xiao-Xia Li, Chen Xin, Brad Kline, Meng-Tian Kang, Meng Li, Li-Ya Qiao, Ning-Li Wang

**Affiliations:** ^1^Beijing Tongren Eye Center, Beijing Tongren Hospital, Capital Medical University, Beijing Ophthalmology and Visual Sciences Key Laboratory, Beijing, China; ^2^Department of Ophthalmology, University of California, San Francisco, San Francisco, CA, United States

**Keywords:** early-onset high myopia, partial cataract, retinal image quality, refractive lens replacement surgery, axial length

## Abstract

**Purpose:**

By reporting clinical characteristics and retinal image quality before and after refractive lens replacement surgery in early-onset high myopia (eoHM) patients presenting with partial cataract, we emphasized the need for an objective way to grade the severity of partial cataracts.

**Methods:**

This retrospective, consecutive case series included six Chinese patients (nine eyes). Analysis of previous medical records, visual acuity, optometry, retinal image quality, and axial length (AXL) before surgery and after surgery was performed.

**Results:**

Five females and one male (nine eyes) with a mean (± SD) age of 11.6 ± 7.9 years (range: 4–25 years) were included in this study. The preoperative spherical power ranged from −7.5 to −42 D. The mean follow-up time was 36 months (range: 24–48 months). Phacoemulsification was followed by in-the-bag implantation of intraocular lens. For patients who were under 6 years old, posterior capsulotomy + anterior vitrectomy were performed simultaneously. All surgeries were uneventful and no postoperative complications occurred during the entire follow-up period. All patients’ uncorrected visual acuity improved by ≥2 lines postoperatively(Snellen acuity). LogMAR best-corrected visual acuity was improved at 24-month (*P* = 0.042) and endpoint (*P* = 0.046) follow-ups. Modulation transfer function cutoff frequency (MTF_cutoff_) and objective scatter index (OSI) was significantly improved at 12-month (*P* = 0.025, *P* = 0.038), 24-month (*P* = 0.005, *P* = 0.007) and endpoint (*P* = 0.005, *P* = 0.008) follow-ups. Postoperative AXL remained stable during 2–4 year follow-ups (*P* > 0.05).

**Conclusion:**

Refractive lens replacement surgery is safe and effective for improving functional vision in eoHM patients presenting with partial cataract. Retinal image quality could provide a useful and objective way to facilitate partial cataract severity evaluation and surgery decision making.

## Introduction

The prevalence of high myopia among young adults is higher among Asian (6.8–21.6%) compared with non-Asian populations (2.0–2.3%) (1). With high myopia becoming a major public vision problem, it is vital to find effective preventive strategies. Patients presenting with refractive errors less than −6.0 D or axial lengths (AXL) larger than 26 mm before school age (7 years old) are defined to have early-onset high myopia (eoHM) (2). It is usually thought to be genetically determined (3, 4). However, there have been a few case reports indicating that childhood cataracts might cause eyeball axial elongation due to form deprivation (5). In our clinic, we found that some eoHM patients were presenting with partial cataract. For children with a partial cataract who can cooperate with the visual acuity test, surgical treatment is warranted if the BCVA is less than 20/50 (6). However, it is believed that the loss of accommodation after the cataract is removed may negatively affect visual function more than the partial cataract itself (6). Moreover, early surgery may also disrupt binocular vision and increase the difficulty of evaluating the dioptric power of the implanted intraocular lens (IOL) (7). Consensus guidelines advise managing affected infants and preschool children by monitoring for possible progression in size or density and pharmacologic pupillary dilation. Extraction is deemed necessary if the cataract progresses to obscure the visual axis or if strabismus or nystagmus develops (8). According to the above criteria, some patients were not considered suitable for cataract surgery. However, they showed myopia aggravation and/or axial elongation during follow-ups. The timing of cataract surgery is one of the main factors influencing visual outcome (9). Each child must be approached individually (6).

Studies have shown that retinal image quality can affect eyeball development. As the second-largest refractive media of the human eye, lenses with different degrees of opacity can cause dispersed light curtain formation before the retina, thus causing retinal image degradation (10). All of our patients were reported to suffer from high myopia at the age before 6 years old. We wonder if partial cataract may be related to retinal image quality degradation at that age and further affect eyeball development. Recently, the Optical Quality Analysis System (OQAS™, Visiometrics SL, Tarrasa, Spain), based on the double-pass technique, was developed to perform an objective evaluation of optical quality. The high sensitivity of this technique in detecting forward scattering makes it a powerful tool to identify earlier stages of cataract and assess for surgery. For age-related cataract, an objective scatter index (OSI) based on the double-pass system >3.0 corresponds to developed cataracts that should undergo surgery (11, 12). However, as far as we know, this technique has not been applied to juvenile cataract.

Herein, we reported six cases of eoHM patients who presented with partial cataract. As retinal image degradation was confirmed by OQAS™, we performed lens extraction combined with IOL implantation for these patients. By comparing visual parameters and retinal image quality before and after surgery, we looked into the effect of partial cataract on retinal image quality and emphasized the need for an objective way to analyze and grade the severity of partial cataracts.

## Materials and Methods

### Patients

This case-series study identified six eoHM patients (nine eyes) from April 2015 to February 2016 with the following inclusion criteria: (1) presented with partial cataract under slit-lamp microscope examination, (2) high myopia (spherical refraction ≤–6.00 D or AXL larger than 26 mm) development before the age of seven, (3) myopia aggravation after ineffective amblyopia treatment (occlusion therapy or amblyopia training) according to previous medical records or self-reported history, and (4) patients without other ocular and systemic disorders based on ophthalmic examinations and preoperative systemic evaluation.

Ethics approval was obtained from the institutional ethic committee, and according to the tenets of the Declaration of Helsinki. Written informed consent was obtained from all patients or their legal guardians.

### Ocular Examinations

All patients were followed-up at 1, 3, and 6 months after refractive lens exchange surgery and at 6- to 12-month intervals thereafter. Regular preoperative and postoperative examinations included slit-lamp microscopy, intraocular pressure, BCVA, funduscopy and optical coherence tomography (OCT). AXL was examined using IOLMaster 500 (Carl Zeiss, Germany) before operation and at each follow-up until the endpoint. All examinations were performed by qualified technicians.

### Optical Quality Parameters Measurement

The retinal image quality of each subject was measured before operation and at each follow-up by OQAS™II, a retinal imaging quality analyzer based on a double-pass system. Patients were asked to blink before the scan to maintain an intact tear film during the scan. In our previous study, no significant difference was found in ocular optical quality parameters obtained using a trial spectacle or the built-in modified Thorner optometer for spherical refractive error correction in the OQAS system (13). Without external optical correction, three different successive independent scans were taken of each eye according to the operation manual by one examiner under dark conditions. Three successive measurements for modulation transfer function cutoff (MTF_cutoff_) and OSI were recorded and the average values were used for further analysis. The MTF represents the loss of contrast produced by the eye’s optics as a function of spatial frequency, which provides information about the overall optical performance of the human eye. The MTF_cutoff_ is the spatial frequency that corresponds to a 0.01 MTF value. A higher MTF_cutoff_ value represents better ocular optical quality. The OSI is calculated by measuring the amount of light outside the retinal point spread function (PSF) image in terms of the intensity of light in the center (14). A higher OSI value represents greater intraocular scatter.

### Cataract Surgery

The SRK/T formula was applied in the preoperative biometry calculation and IOL power calculation (15). Standard phacoemulsification was performed by experienced surgeons (BY and LY Q) through a 3.2 mm clear cornea tunnel incision without suture. Phacoemulsification was followed by in-the-bag implantation of IOL, which was a single-focus aspheric hydrophobic soft lens. For Case 1 and Case 2, central capsulorhexis of the posterior capsule and anterior vitrectomy were performed after IOL implantation. Patients were examined daily in the first 2 days after the operation and then weekly for the first month. Then, they were followed up at 3 and 6 months and at 6–12 months intervals thereafter. Case 3, 5, and 6 (four eyes) underwent YAG-laser capsulotomies at 12–18 months postoperatively (Table 1).

**TABLE 1:** Clinical characteristics and retinal image quality by case number and operative eye of patients, organized by age at the onset.

Case No.	Age at the onset (y)	Previous sphere diopter (D)	Age at surgery (y)	Surgery	Pre-op Sphere diopter (D)	Follow-up time (month)	Pre-op BCVA	Endpoint BCVA	Pre-op MTF_outoff_ (cpd)	Endpoint MTF_outoff_ (cpd)	Pre-op OSI	End-point OSI	Pre-op AXL (mm)	Endpoint AXL (mm)
1 Right	2	NA	4	Phaco + IOL + central capsulorhexis + anterior vitrectomy	−9	48	0.16	1.0	1.54	37.08	8.7	2.3	24.4	26.76
2 Left	3	−5, −8.75	5	Phaco + IOL + central capsulorhexis + anterior vitrectomy	−10	48	NA	0.6	3.04	17.50	10.3	3.9	24.2	25.34
3 Right	3	−26.5, −29, −25	11	Phaco + IOL	−42	36	0.05	0.5	5.03	44.77	9.1	1	32.44	32.54
3 Left	3	−12, −15, −18	11	Phaco + IOL(YAG for PCO at 1 year)	−26.5	36	0.16	0.5	9.31	45.14	8.6	1	29.17	29.32
4 Right	5	−8.75	7	Phaco + IOL	−10	36	0.6	0.8	14.30	18.25	6	4	27.34	29.22
4 Left	5	−10.5	7	Phaco + IOL	−12	36	0.6	0.9	9.24	20.93	7.8	3.1	28.1	29.95
5 Left	5	NA	25	Phaco + IOL(YAG for PCO at 1.5 year)	−15.5	36	0.7	1.0	11.16	40.95	3.6	0.8	26.51	26.43
6 Right	5	NA	15	Phaco + IOL(YAG for PCO at 1.5 year)	−19	36	0.4	1.0	9.65	39.68	3.2	0.8	27.77	28.07
6 Left	5	NA	15	Phaco + IOL(YAG for PCO at 1.5 year)	−21	24	0.3	1.0	7.35	31.98	4.7	1.1	28.41	28.78

*Pre-op, preoperative; D diopter, BCVA best-corrected visual acuity (presented as Snellen decimal visual acuity); AXL, axial length; MTF_cutoff_, modulation transfer function cutoff frequency; cpd, cycles per degree; OSI, objective scatter index; N, not applicable; YAG, yttrium–aluminum–garnet laser; PCO, posterior capsular opacity; Phaco, phacoemulsification; IOL, intraocular lens implantation.*

### Statistical Analyses

Statistical analysis was performed with SPSS software for Windows (version 20.0 SPSS, Inc, Chicago, IL, United States). The normality of the data distribution was assessed by the Kolmogorov–Smirnov test, and a *P*-value >0.05 was considered normally distributed. Boxplot was applied to check for outliers. Descriptive analyses were performed to compute the mean and standard deviation in preoperative and postoperative outcome measures. Data that did not show normality was presented as median (interquartile range, IQR). All visual acuity measurements were converted to the logarithm of the minimum angle of resolution (LogMAR) visual acuity before statistical analysis, but presented in Table 1 as Snellen decimal visual acuity for easy understanding. The one-way repeated measures ANOVA was used to compare preoperative and postoperative LogMAR BCVA, MTF_cutoff_, OSI, and AXL. Mauchly’s test of sphericity was applied to check the variance-covariance matrix of the dependent variable. If Mauchly’s test of sphericity was not met, epsilon correction was further applied using the Greenhouse-Geisser method, and partial η2 was reported. All tests were considered to be statistically significant if *P* < 0.05.

## Results

**FIGURE 1:**
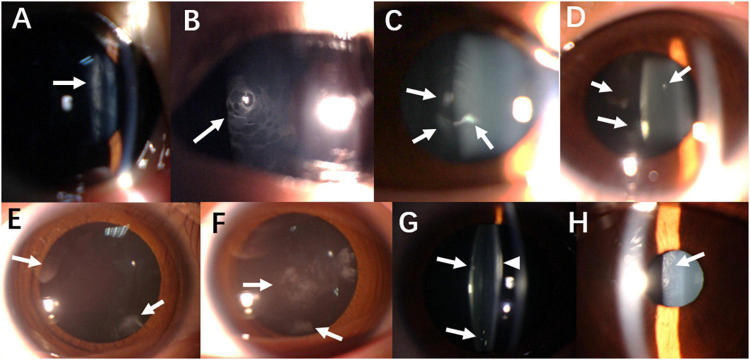
Slit-lamp biomicroscopy photography showing lens opacity characteristics. **(A)** Cortex mass opacity (arrow) in the right eye of Case 1. **(B)** Non-uniformed rarefied cloudy opacity of posterior capsule (arrow) in the left eye of Case 2. **(C)** Posterior capsule opacity (arrow) in the right eye of Case 3. **(D)** Similar finding (arrow) was observed in the left eye of Case 3. **(E)** Posterior sub-capsule opacity (arrow) in the right eye of Case 4. **(F)** Similar finding (arrow) was observed in the left eye of Case 4. **(G)** Snowflake-like cortex opacity (arrow) and evenly condensed cortex (arrowhead) in the left eye of Case 5. **(H)** Rarefied cloudy opacity of posterior capsule (arrow) in both eyes in Case 6.

In this case series study, five patients were female (seven eyes) and one was male (two eyes). For the contralateral eyes that were excluded from this study, one was emmetropia and the other two eyes were less than −6D. The mean (± SD) age was 11.16 ± 7.9 years (range: 4–25 years). Slit-lamp microscopy showing partial cataract morphology of each patient is presented in Figure 1. The preoperative spherical power ranged from −7.5 D to −42 D. Tessellated fundus with peripapillary atrophy, without peripheral degeneration, was noted in the study eyes preoperatively. Also, OCT showed normal macula structure, without macular schisis or atrophy. Postoperatively, two eyes showed wider peripapillary atrophy, and one eye’s fundus photography was clearer after lens extraction. The mean follow-up time was 36 (Q1-Q3: 36–36) months (range: 24–48 months). The characteristics of patients’ preoperative data, follow-up time, and endpoint data for BCVA, MTF_outoff_, OSI, and AXL are presented in Table 1. In case 2 and case 3, high myopia progression was shown according to the previous medical record. A family history of high myopia was also collected. For case 1, the patient’s father suffered from congenital cataract, high myopia of both eyes, and retinal detachment of the left eye. The father of case 6 also suffered from high myopia. All surgeries were uneventful and no postoperative complications occurred during the entire follow-up period such as retinal detachment or secondary glaucoma.

Patients’ refractive and uncorrected visual acuity (UCVA) outcomes are presented in Table 2. IOL was selected to achieve a goal refraction of + 2 D in cases 1 and 2, whose ages were 4 and 5 years old, respectively. For case 4, whose age was 7 years old, goal refraction was + 0.5D. In the remaining cases, the goal refraction was plano to −2 D, according to their life need. For case 6, one eye was designed to be nearsighted, and the contralateral eye emmetropia. Initial spherical correction (treatment achieved) averaged 18.25 ± 11.15 D (range: 7.75–44.0 D). Five eyes (56%) were corrected to within ± 1.0 D of spherical goal refraction. Of the remaining four eyes, two were under-corrected (residual myopia of −1.25 and −2.25 D) and two were overcorrected (0 and + 2 D). Hyperopic and myopic shift (regression) during the follow-up interval is reported in Table 2 as a regression rate, expressed as (final postop refraction - initial postop refraction)/follow-up time. Eight eyes (89%) exhibited myopia shift (∼−0.5 D/year except for case 3), whereas case 5 (age 25) showed hyperopic change.

**TABLE 2:** Refractive and uncorrected visual acuity outcomes by case number and operative eye of patients, organized by age at the onset.

Case No.	Pre-op Sphere diopter (D)	Goal Refraction (D)	Initial postop Refraction (D)	Spherical correction achieved (D)	Final postop refraction (D)	Follow-up (years)	Regression (D/year)	Preop cylinder (D)	Initial postop cylinder (D)	Final postop cylinder (D)	Preop UDVA	Postop UDVA
1 Right	−9	1.64	−1.25	7.75	−4	4	−0.68	1.5	1.25	0.5	0.1	0.3
2 Left	−10	1.91	1	11	−1.3	4	−0.57	1	1	0.75	NA	0.5
3 Right	−42	−2.15	2	44	−2	3	−1.3	2	1.5	1.5	0.03	0.3
3 Left	−26.5	−2.18	0	26.5	−3	3	−1.0	3.5	3.5	3.5	0.04	0.3
4 Right	−10	0.38	1.5	11.5	−0.5	3	−0.67	1.75	3	1.25	0.1	0.5
4 Left	−12	0.54	0.75	12.75	−0.5	3	−0.41	1.75	2	1	0.1	0.6
5 Left	−15.5	−0.38	−2.25	13.25	−1.5	3	0.25	4.5	3.5	3.5	0.04	0.6
6 Right	−19	−0.65	−0.5	18.5	−1.5	2	−0.5	1	1.5	1.5	0.1	0.7
6 Left	−21	−1.97	−2	19	−3	2	−0.5	1	1	1	0.1	0.4

*Pre-op, preoperative; D, diopter; UCVA, uncorrected visual acuity (presented as Snellen decimal visual acuity).*

All nine eyes had some degree of astigmatism before surgery. Post-operatively, cylinder remained unchanged in two eyes, diminished in six eyes, and increased by 0.5 D in one eye. All patients’ UCVA improved ≥2 lines postoperatively. Due to residual myopia and astigmatism, BCVA was used for comparison and analysis.

**FIGURE 2:**
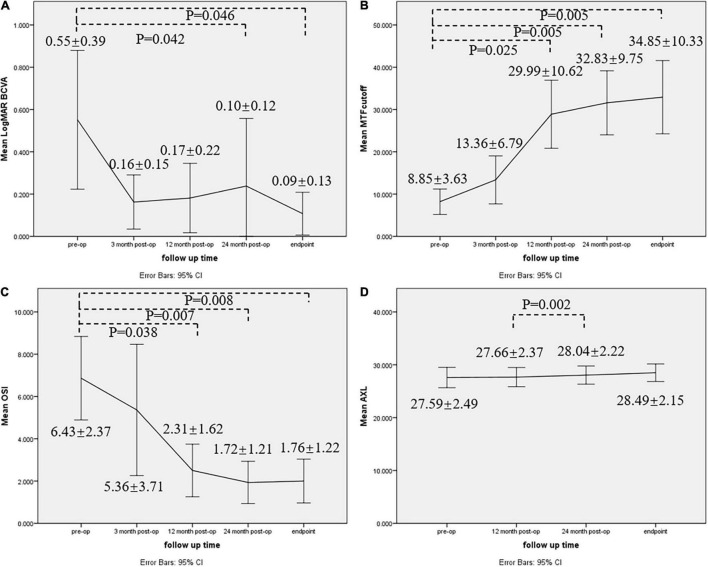
Line graph showing preoperative and postoperative comparisons for **(A)** LogMAR BCVA, **(B)** MTF_cutoff_, **(C)** OSI, and **(D)** AXL. Mean ± SD of each parameter was shown, and *P*-value was displayed if the change was significant (*P* < 0.05). BCVA best-corrected visual acuity, AXL axial length, MTF_cutoff_ modulation transfer function cutoff frequency, OSI objective scatter index.

Logarithm of the minimum angle of resolution BCVA, MTF_cutoff_, and OSI at pre-operative, 3 months, 12 months, 24 months, and endpoint were compared using one-way repeated measures of ANOVA, as well as AXL at preoperative, 12 months, 24 months, and endpoint. A line graph was drawn to show the mean ± SD of each parameter at each follow-up time (Figure 2). *P*-value was displayed if the change was significant (*P* < 0.05).

Logarithm of the minimum angle of resolution BCVA was statistically significant among different time points (F (1.611, 11.276) = 12.948, *P* = 0.02, partial η2 = 0.649). LogMAR BCVA was improved at 24-month (95%CI: 0.015–0.878, *P* = 0.042) and endpoint (95%CI: 0.008–0.909, *P* = 0.046) follow-ups, but the improvement was not significant at 3-month (95%CI: −0.019–0.797, *P* = 0.063) and 12-month (95%CI: −0.016–0.787, *P* = 0.062) follow-ups. No significant difference was found among post-operative follow-ups (*P* > 0.05).

Modulation transfer function cutoff frequency was significantly improved at 12-month (95%CI: −39.692–2.591, *P* = 0.025), 24-month (95%CI: −39.976–7.983, *P* = 0.005) and endpoint (95%CI: −43.221–8.782, *P* = 0.005) follow-ups, but the improvement was not significant at 3-month (95%CI: −14.695–5.663, *P* = 1.000) follow-up. No significant difference was found among post-operative follow-ups (*P* > 0.05).

Objective scatter index was statistically significant among different time points *F*(1.380, 9.662 = 9.790, *P* = 0.008, partial η2 = 0.583). OSI was significantly improved at 12-month (95%CI: 0.217–8.033, *P* = 0.038), 24-month (95%CI: 1.442–7.983, *P* = 0.007) and endpoint (95%CI: 1.315–8.035, *P* = 0.008) follow-ups, but the improvement was not significant at 3-month (95%CI: −3.040–5.190, *P* = 1.000) follow-up. No significant difference was found among post-operative follow-ups (*P* > 0.05).

Axial length was statistically significant among different time points *F*(1.151, 9.205 = 8.893, *P* = 0.013, partial η2 = 0.526), but postoperative AXL at all follow-up times did not change significantly compared to pre-operative status (*P* > 0.05). However, AXL at 24 months was significantly longer than AXL at 12 months (95%CI: −0.607–0.153, *P* = 0.002).

## Discussion

Our surgical interventional case series study showed that six eoHM patients (nine eyes) presenting with partial cataract showed improvement of BCVA and retinal image quality after refractive lens exchange surgery. Moreover, AXL remained stable during 2–4 year follow-ups.

Patients in our study presented with the following characteristics: (1) early-onset high myopia with degraded BCVA, (2) ineffective amblyopia treatment before surgery, (3) myopia aggravation and axial length elongation before surgery according to previous medical records or self-report, and (4) partial cataract and optical quality degradation according to OQAS. We speculated that partial cataract may increase forward scatter in the eye, causing retinal image degradation and incomplete form-deprivation, even though the lens opacity was off the optic axis. Since childhood form deprivation caused by cataract could cause AXL elongation, we suspected that partial cataract in our patients may cause paracentral visual disturbance and impact vision development (5).

The scattering of the eye can be divided into light scattered toward the retina (forward scatter) and light scattered backward (backscatter). Forward scatter has been well recognized to have functional importance, and the double-pass technique is proposed to estimate the effect of forward scattering on vision (16). High myopia can cause significant retinal image degradation. Mean (± SD) MTF_cutoff_ was reported to be 32.38 ± 9.73 and 27.61 ± 8.11 cpd in the high myopia group (−6 D> spherical equivalent refraction ≥−9 D) and super-high myopia group (spherical equivalent refraction <−9 D). Mean (± SD) OSI was 0.89 ± 0.61 and 1.33 ± 0.65 in the high and super-high myopia groups (17). Optical quality in the younger population is supposed to be better than that in the older population (18). Although the normal value of optical parameters in healthy children under 10 years old has not been reported, the mean (± SD) MTF_cutoff_ value in ages 10–15 of the normal population is 46.85 ± 7.45 cpd, while the mean (± SD) OSI value is 0.34 ± 0.22 (18). For healthy adults 18–30 years old, the mean MTF_cutoff_ and OSI are 44.54 cpd and 0.38, respectively (19). In our case series, with mean (± SD) age of 11.16 ± 7.9 years(range: 4–25 years), the mean (± SD) preoperative MTF_cutoff_ and OSI were 7.85 ± 4.04 cpd (range: 1.54−14.31) and 6.88 ± 2.58 (range: 3.2−11.9), respectively. It was obvious that the preoperative optical quality of our patients was significantly worse than age- and myopia-matched non-cataract individuals.

The optimal timing of surgery for pediatric partial cataract is often problematic. Very early surgery is indicated in cases of dense bilateral congenital cataract. A moderately cloudy unilateral cataract also needs a more aggressive approach (20). Proper visual stimulation in early childhood is critical to the development of normal vision (21). It is obvious that a judgment based on varied cataract morphology is quite subjective and equivocal.

Objective scatter index has already been reported to be a useful parameter to objectively grade age-related cataracts in elderly patients and preoperative evaluation of patients with early cataract (12, 22). OSI values between 3 and 7 corresponded to developed cataracts that should undergo surgery. OSI values higher than 7 were seen in eyes with severe cataracts (12). In our case series, if the same criteria could be applied in pediatric partial cataract assessment, our patients should have undergone surgery earlier. However, as far as we know, no study has reported objective grading criteria for partial cataract yet. By reporting this group of patients, we want to emphasize the importance of an objective method for pediatric partial cataract severity evaluation and surgery decision making.

Many refractive surgery techniques have been applied to treat pediatric large magnitude ametropia and anisometropia, especially in children who are non-compliant with spectacle wear or with neurodevelopmental disorders (23–25). LASEK (laser-assisted subepithelial keratectomy)/photorefractive keratectomy (PRK), lensectomy or refractive lens exchange, and intraocular collamer lens (Visian ICL) implantation can achieve comparable acuity gains according to previous reports (24, 26). With the maturity of cataract surgery equipment and technique, considering that our patients have partial cataract, we employed refractive lens exchange surgery (for younger patients who cannot cooperate with postoperative laser capsulotomy, posterior capsulotomy + anterior vitrectomy was performed simultaneously). Secondary glaucoma, retinal detachment, or other complications did not occur in the follow-up, which proved that the procedure is relatively safe and applicable.

All patients achieved favorable results in this pilot study, with increased UCVA and BCVA, better retinal imaging quality, and relatively stabilized AXL. AXL in four eyes increased by more than 1 mm at the endpoint follow-up. One reason was that their age was under 7 years old (4,5, and 7, respectively). AXL growth of approximately 1 mm per year is a part of developmental process (27). The mean regression and AXL growth in our patients were comparable with previous studies, and regression is due mainly to increasing length, at least in younger children (24, 27). Notably, improvement of BCVA and optical quality occurred 12 months postoperatively (Figure 2). It is reasonable to think that, apart from refractive correction that may cause immediate visual improvement through image magnification, retinal image clarity may also contribute to gradual visual development in our patients, especially in children. This implied that retinal image quality degradation caused by partial cataract may be related to the occurrence and development of high myopia. Removing lens opacity may be an effective intervention for eoHM prevention and myopia progression control.

The current study had several limitations. Firstly, the connection between partial cataract and retinal image degradation needs further investigation. In a future project, we will compare retinal image quality in high myopia patients with or without partial cataract. By using contact lenses to correct high myopia in patients with partial cataract, we can further distinguish whether retinal image degradation was caused by high myopia or partial cataract. Secondly, due to the retrospective nature of the study, preoperative visual acuity in case 1 was not recorded due to bad compliance owing to the patient’s young age. A preferential looking test should be applied in such a situation. Thirdly, data from paired eyes are likely to be correlated. However, in populations with asymmetric eye disease, the use of data from both eyes is wholly appropriate (28). Considering that we recruited myopes rather than healthy individuals, using data from both eyes seems acceptable. Fourthly, eoHM present before school age is likely to be determined by genetic defects (3, 4). However, mutations in these genes have been identified in only a few families with eoHM (29). In our case series, two patients (Case 1 and Case 6) had a family history of high myopia. Further investigation is needed to verify whether genetic factors played a role in the pathogenesis of our patients’ disease. Lastly, young patient age and high myopia were both risk factors for pseudophakic retinal detachments (30). Fortunately, no postoperative complications occurred during the entire follow-up period in our patients. As high myopia was irreversible, doctors should avoid posterior capsular rupture during the surgery and inform the patients to avoid eye trauma.

## Conclusion

Our surgical interventional case series study found that refractive lens replacement surgery is safe and effective for improving functional vision in eoHM patients presenting with partial cataract. Retinal image quality could provide a useful and objective way to facilitate partial cataract severity evaluation and surgery decision-making.

## Data Availability Statement

The raw data supporting the conclusions of this article will be made available by the authors, without undue reservation.

## Ethics Statement

The studies involving human participants were reviewed and approved by the institutional Ethics Committee of Beijing Tongren Hospital, Capital Medical University. Written informed consent to participate in this study was provided by the participants’ legal guardian/next of kin.

## Author Contributions

X-FZ was involved in the design of the work, data collection, data analysis, and drafting the article. X-XL and CX were involved in data analysis, interpretation, and critical revision of the article. BK and M-TK were involved in data analysis and interpretation. ML were involved in data collection and data interpretation. L-YQ was involved in the design of the work, data analysis, critical revision, and final approval of the article to be published. N-LW was involved in the conception of the work and interpretation. All authors read and approved the final manuscript.

## Conflict of Interest

The authors declare that the research was conducted in the absence of any commercial or financial relationships that could be construed as a potential conflict of interest.

## Publisher’s Note

All claims expressed in this article are solely those of the authors and do not necessarily represent those of their affiliated organizations, or those of the publisher, the editors and the reviewers. Any product that may be evaluated in this article, or claim that may be made by its manufacturer, is not guaranteed or endorsed by the publisher.

## Abbreviations

eoHM: early-onset high myopia
AXL: axial length
MTF_cutoff_: modulation transfer function cutoff frequency
OSI: objective scatter index
BCVA: best-corrected visual acuity
IOL: intraocular lens
OQAS: Optical Quality Analysis System
PSF: point spread function
IQR: interquartile range
LASEK: laser-assisted subepithelial keratectomy
PRK: photorefractive keratectomy
UCVA: uncorrected visual acuity
Pre-op: preoperative
D: diopter
cpd: cycles per degree
YAG: yttrium–aluminum–garnet laser
PCO: posterior capsular opacity
Phaco: phacoemulsification.
